# Hydrogen Bonding in Human P450-Substrate Interactions: A Major Contribution to Binding Affinity

**DOI:** 10.1100/tsw.2004.210

**Published:** 2004-12-10

**Authors:** David F.V. Lewis

**Affiliations:** School of Biomedical and Molecular Sciences, University of Surrey, Guildford, Surrey, GU2 7XH, UK

**Keywords:** hydrogen bonding, cytochrome P450, enzyme-substrate binding, interactions

## Abstract

The importance of hydrogen bonding, a relatively strong intermolecular force of attraction between molecules in biological systems, is discussed in the respect of P450 substrate affinity towards one or more of the human P450 enzymes that are generally associated with drug and other xenobiotic metabolism. It is shown that calculation of hydrogen bond distances and energies based on simple empirical relationships provide values that agree closely with experimental findings. It is thus possible to estimate the hydrogen bond contribution to P450 enzyme-substrate binding affinity based on modelled interactions and by use of these relatively simple formulae, particularly when employed in conjunction with substrate-lipophilicity relationships.

## INTRODUCTION

The cytochromes P450 (CYP) constitute a superfamily of heme-thiolate enzymes that have been reported in all biological kingdoms and in most species. Although over 2700 individual P450s have been sequenced, there are only about 10 unique high-resolution crystal structures currently available to investigate various aspects of P450 structure and function. However, it has been proved possible to derive homology models of many P450 enzymes from appropriate crystallographic templates and, for human P450s, the recently reported rabbit CYP2C5 structure appears to be of particular benefit. In many of the P450 structures investigated thus far, including both models and crystallographic coordinates, the ubiquitous importance of hydrogen bonding between enzyme and substrate has been emphasized. Consequently, it can be assumed that this is one of the major contributions to the overall binding energy between substrate and enzyme in P450-substrate interactions. Evidence for this comes from the location of potential hydrogen bond acceptors and donors in P450 crystal structures. For example, in the substrate-bound CYP101 structure, the substrate camphor is positioned directly above the heme moiety for 5-exo hydroxylation by apparently forming a hydrogen bond with the tyrosine-96 residue. There are also several hydrophobic residue contacts that cooperatively assist in the binding and orientation of the camphor substrate. In fact, a simple calculation based on compound lipophilicity and average hydrogen bond energy gives a good estimate of the camphor substrate binding energy of CYP101, which is in satisfactory agreement with the experimentally determined value[1].

## Estimation of Hydrogen Bond Lengths and Energies

However, it is apparent from a survey of the literature that hydrogen bond energies can vary quite significantly depending on the nature of the electronegative atoms involved in the hydrogen bond formation itself[2,3,4,5,6,7,8,9,10,11]. For example, Table 1 provides a summary of the major types of hydrogen bonds that are likely to be encountered in biological systems. Interestingly, one can obtain a satisfactory estimate of the hydrogen bond length from a sum of the individual heavy atom van der Waals Radii[12]. In fact, the correlation between calculated and observed hydrogen bond lengths is very good (R = 0.98) as shown in Table 1, and Fig. 1 provides a graphical comparison of these data. Consequently, this finding can be employed in the calculation of hydrogen bond energies for systems where the actual bond length may not be available. Table 2 represents a summary of average bond lengths for the most common hydrogen bonds and the hydrogen bond distances encountered in proteins, resulting from side-chain interactions, is also provided showing that these values tend to mirror those found in other systems. It would, therefore, be straightforward to use this information for calculating hydrogen bond lengths in enzyme-substrate interactions.

An empirical relationship between hydrogen bond energy, $HB_e$, and the inverse of distance has been developed[8], which includes consideration of the group dipole moment, $\mu_{XH}$, and difference in ionization potential, $\Delta I_Y$, for the components in a hydrogen bond XH···Y, as follows:

$$HB_e = -1.64\ \mu_{XH}\ \Delta I_Y / r_{xy}$$

where $r_{xy}$ is the hydrogen bond distance between the two hetero atoms X and Y, which make up the hydrogen bond. Using this equation, it is possible to derive calculated values for hydrogen bond energies that agree closely with experimental data. Table 3 shows the relevant set of values for a number of typical hydrogen bonds mentioned previously. Although some of the hydrogen bonds involving sulfur give values lower than the experimental energies, the remaining nine bond types are satisfactorily reproduced by the above expression. The correlation equation shown in Table 3 has a slope which is very close to unity (actually 1.04) and the correlation coefficient (R value = 0.98) is excellent for nine points; whereas Fig. 2 shows a comparison between experimental hydrogen bond energies and calculated values. Although two hydrogen bond types were clear outliers, these were both involving sulfur as a hydrogen bond acceptor and it is possible that the ionization energy parametrization requires modification to improve the accuracy of bond energy calculations for such hydrogen bond atom types as sulfur, for example. Nevertheless, the concordances are satisfactory for those cases where sulfur acts as a hydrogen bond donor to nitrogen and oxygen, as shown in Table 3.

Consequently, it is reasonable to make use of the empirical relationship described above for the calculation of any hydrogen bond energies likely to be encountered in P450-substrate interactions, such that a more precise estimate of the hydrogen bond contribution to the overall binding affinity can be achieved. However, the employment of an average value of –2 kcal/mol for a hydrogen bond does appear to give rise to satisfactory results in the analysis of such interaction energies[13]. It should be noted that a correction is made for the local dielectric constant of the binding site, which is generally assumed to be approximately 4. Therefore, it would be necessary to make a small modification to the Allen relationship, described previously, to account for the local dielectric constant of the binding site. An amended form of this expression would, therefore, be of the form:

$$HB_e = -0.41\ \mu_{XH}\ \Delta I_y / r_{xy}$$

**TABLE 1**
**Typical Hydrogen Bond Energies and A-H...B Distances in Various Systems[4,12,14,15,16]**

| | Hydrogen Bond Type | ΔH ( kcal/mol) | Average Distance (Å) | Sum of A...B Radii (Å) |
|---|---|---|---|---|
| 1. | $O-H...O=C\langle$ | –4.66 | 2.76 | 2.80 |
| 2. | $O-H...O\langle$ | –5.02 | 2.82 | 2.80 |
| 3. | $O-H...N\Xi$ | –7.65 | 2.88 | 2.90 |
| 4. | $O-H...N\langle\!\!\langle$ | –6.45 | 2.88 | 2.90 |
| 5. | $N-H...O=C\langle$ | –3.47 | 2.90 | 2.90 |
| 6. | $N-H...O\langle$ | –2.99 | 2.90 | 2.90 |
| 7. | $N-H...N\langle\!\!\langle$ | –3.59 | 3.10 | 3.00 |
| 8. | $O-H...F-$ | –3.70 | 2.72 | 2.75 |
| 9. | $O-H...Cl-$ | –2.20 | 3.12 | 3.20 |
| 10. | $O-H...S\langle$ | –4.18 | 3.31 | 3.25 |
| 11. | $N-H...S\langle$ | –3.59 | 3.44 | 3.45 |
| 12. | $S-H...N\langle\!\!\langle$ | –2.39 | 3.44 | 3.45 |
| 13. | $N-H...F$ | –3.70? | 2.78 | 2.85 |
| 14. | $S-H...O$ | –2.00 | 3.31 | 3.25 |
| 15. | $F-H...F$ | –6.80 | 2.55 | 2.70 |

… Denotes a hydrogen bond.

? Refers to an estimated value for the hydrogen bond energy based on other reported data for hydrogen bond energies in the literature.

**van der Waals radii:** Atom, Radius (Å); O, 1.40; N, 1.50; S, 1.85; Cl, 1.80, F, 1.35.

The agreement between experimental and calculated hydrogen bond distances is very good (R = 0.98) indicating that the sum of the heavy atom van der Waals radii is a satisfactory estimate of actual hydrogen bond distances observed in crystal structures, thus providing a method for calculating hydrogen bond distances in general.

which simply involves division of the constant term by the dielectric constant of the medium, assumed to have a value of 4 at the center of globular proteins. This expression could then be used with some degree of confidence in the estimation of likely hydrogen bond energies encountered in P450-substrate binding interactions.

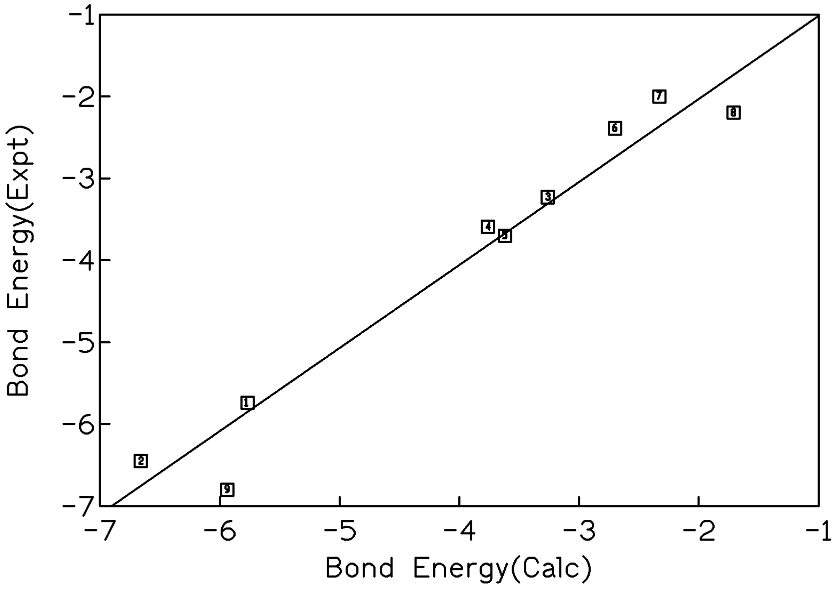

**FIGURE 1.** Comparison between calculated and experimental hydrogen bond lengths using data presented in Table 1.

## Hydrogen Bonding and P450-Substrate Binding Affinity

Although hydrogen bonds play an important role in many aspects of biological activity at the molecular level, it is clear from extensive structural studies on cytochrome P450 that usually at least one hydrogen bond is formed between the substrate and enzyme active site, resulting in orientation of the former for metabolism at a known position (reviewed in Lewis[17,18]). For example, coumarin is orientated for 7-hydroxylation by the human enzyme CYP2A6 via a hydrogen bond with an asparagine residue close to the heme moiety within the active site region (Fig. 3). Clearly, this hydrogen-bonding element represents a major contribution to the overall binding affinity, although desolvation of the enzyme's active site also plays an important role[13]. The free energy of binding for coumarin is about –8 kcal/mol with the lipophilicity component comprising approximately –2 kcal/mol, and there is a similar contribution from aromatic $\pi$-$\pi$ stacking. Consequently, the hydrogen-bonding component for this interaction may consist of two hydrogen bonds, of energy approximating to –2 kcal/mol for each hydrogen bond. This finding agrees closely with the typical average value for hydrogen bonds in biological systems, which were discussed previously, where a value of –2 kcal/mol probably represents a reasonable estimate for an average hydrogen bond energy in a globular protein where the local dielectric constant is likely to be about 4, as shown in Table 4.

Although water is present in the environment, even within hydrophobic regions of the protein interior, it is generally accepted that the dieletric constant of the medium will be considerably less than that of pure water, which has a dielectric constant of about 78. In fact, the region at the protein surface has had its dielectric constant estimated at roughly half the value for pure water, and it can be expected that this would diminish markedly for the hydrophobic interiors of globular proteins, despite the fact that some water molecules are likely to be present. However, in many P450-substrate interactions, most of this bound water will be removed following the binding of substrate, in accordance with an entropy-driven desolvation process.

**TABLE 2**
**Hydrogen Bond Distances in Chemical and Biological Systems[4,19]**

| Average Bond Lengths for Hydrogen Bonds (Å) | |
|---|---|
| O–H --- O | 2.70 |
| O–N --- N | 2.85 |
| N–H --- O | 2.90 |
| N–H --- N | 3.05 |
| O–H --- S | 3.31 |
| N–H --- S | 3.39 |
| O–H --- Cl | 3.20 |
| N–H --- Cl | 3.30 |
| N–H --- F | 3.85 |
| F–H --- F | 2.70 |
| **Hydrogen Bond Distances in Proteins Involving Amino Acid Side Chains (Å)** | |
| Ser | 3.0 |
| Thr | 2.9 |
| His | 3.0 |
| Trp | 3.0 |
| Lys | 2.9 |
| Arg | 3.0 |
| Asn | 3.0 |
| Gln | 3.0 |
| Asp | 2.9 |
| Glu | 2.9 |
| Cys | 3.5 |
| Met | 3.4 |

--- Denotes a hydrogen bond.

In general, this desolvation component to the overall substrate binding energy tends to make the major contribution, although a degree of hydrogen bonding is often present. This is because a substantial proportion of P450 substrate possesses hydrogen bond donor/acceptor atoms that are able to form hydrogen-bonded interactions with complementary amino acid residues in the vicinity of the enzyme active site. Based on the generally held view that binding interactions between substrates and P450 enzymes are made up primarily from a combination of hydrogen bonding and π-π stacking energies, together with a log P-related desolvation component, it has been possible to estimate overall binding energies that agree closely with experimental determinations[13]. The correlation between calculated and experimental binding energies is as high as 0.98 for 90 compounds, and the binding affinity for a test set of 10 compounds also gave very good agreement with experimental values[13]. Interestingly, a QSAR study on the same group of substrates gave rise to a regression equation that supported the use of –2 kcal/mol for an average hydrogen bond, thus providing support for this type of approach[13]. Consequently, it is expected that, at least for P450-substrate interactions, the value of –2 kcal/mol represents a reasonable estimate for an average hydrogen bond energy.

**TABLE 3**
**Comparison between Experimental and Calculated Hydrogen Bond Energies[4,8,12]**

| System | | $R^{vdw}_{xy}$ | $\mu_{XH}$ | $\Delta I_Y$ | $E^{calc}_{HB}$ | $E^{expt}_{HB}$ |
|---|---|---|---|---|---|---|
| 1. | OH ··· O | 2.80 | 1.13 | 8.94 | –5.77 | –5.74 |
| 2. | OH ··· N | 2.90 | 1.13 | 10.68 | –6.66 | –6.45 |
| 3. | NH ··· N | 2.90 | 0.66 | 8.94 | –3.26 | –3.23 |
| 4. | NH ··· N | 3.00 | 0.66 | 10.68 | –3.76 | –3.59 |
| 5. | OH ··· F | 2.75 | 1.13 | 5.51 | –3.62 | –3.70 |
| 6. | OH ··· S | 3.25 | 1.13 | 5.29 | –2.94 | –4.18 |
| 7. | NH ··· S | 3.35 | 0.66 | 5.29 | –1.67 | –3.59 |
| 8. | SH ··· N | 3.35 | 0.53 | 10.68 | –2.70 | –2.39 |
| 9. | SH ··· O | 3.25 | 0.53 | 8.94 | –2.33 | –2.00 |
| 10. | OH ··· Cl | 3.20 | 1.13 | 3.02 | –1.71 | –2.20 |
| 11. | FH ··· F | 2.70 | 1.82 | 5.51 | –5.94 | –6.80 |

··· Denotes a hydrogen bond.

Bond types 6 and 7 have been omitted from the following correlation equation. This would appear to be due to an underestimation of the experimental values, possibly because of a relatively low $\Delta I_Y$ energy for sulfur when acting as a proton acceptor.

$$E^{expt}_{HB} = \underset{(\pm 0.079)}{1.014}\, E^{calc}_{HB} \qquad n = 9;\ s = 0.3927;\ R = 0.98;\ F = 171.54$$

n = number of points, s = standard error, R = correlation coefficient, F = variance ratio.

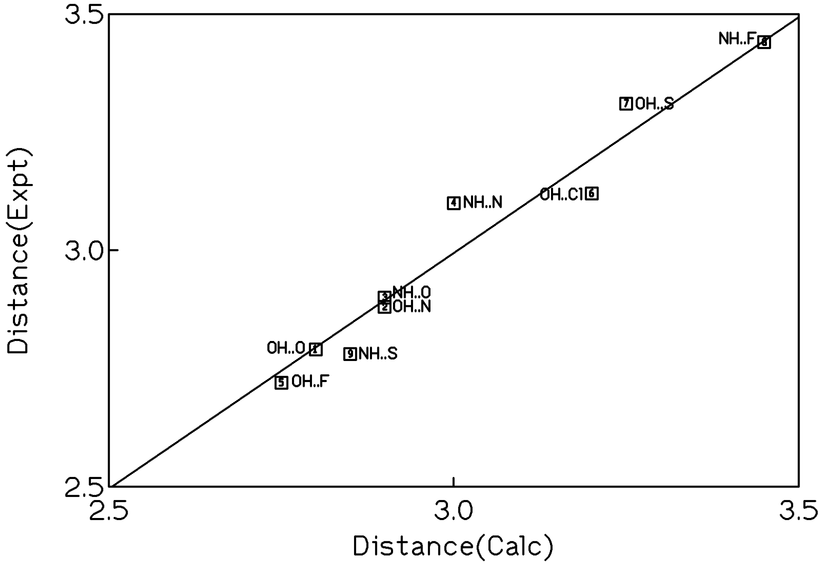

**FIGURE 2.** Comparison between calculated and experimental hydrogen bond lengths using data presented in Table 3.

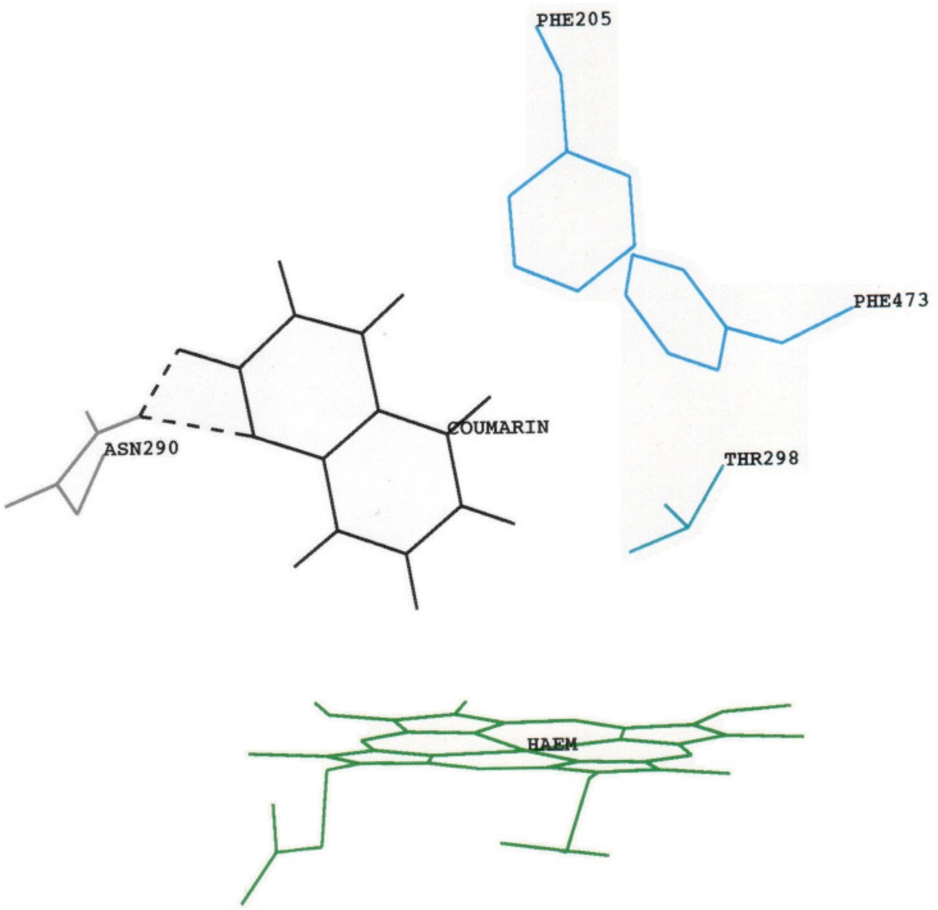

**FIGURE 3.** Putative active site interaction between coumarin and CYP2A6, which metabolizes this compound to 7-hydroxycoumarin. Hydrogen bond interactions between the substrate and enzyme are shown as dashed lines, and displayed amino acid residues are labeled, together with the heme (haem) moiety and coumarin substrate.

## CONCLUSIONS

Hydrogen bonding plays an important role in P450-substrate interactions. This is in terms of contribution to the overall binding affinity and in providing a support for substrate recognition and orientation of metabolism. This is achieved by anchoring the substrate molecule relative to the heme iron such that it may become oxygenated in a certain position. Therefore, it is possible to estimate the overall binding energy between substrate and P450, based on the modelled interaction, by using average values for hydrogen bond energies and other contributions. However, a more precise treatment of hydrogen bonding should give rise to improved predictions of P450-substrate binding energies, and this approach may be more widely applicable to other ligand-binding or substrate-binding interactions in biological systems.

## ACKNOWLEDGMENT

The financial support of GlaxoSmithKline Research & Development Limited, Merck, Sharp & Dohme Limited and the University of Surrey is gratefully acknowledged.

**TABLE 4**
**Hydrogen Bond Energies in Various Chemical Systems[14]†**

| | Donor | Acceptor | ΔH (kJ/mol) | ΔH (kcal/mol) | Average Value (kcal/mol) |
|---|---|---|---|---|---|
| **Oxygen-Oxygen** | | | | | |
| O - H...O = C/\ | Phenol | Acetone | –19.5 | –4.66 | –4.64 kcal/mol |
| | Cholesterol | Glyceryltriacetate | –18.0 | –4.30 | (–2.3) |
| O - H...O/\ | Water | Dioxan | –14.5 | –3.47 | –4.64 kcal/mol |
| | Phenol | Dioxan | –21.0 | –5.02 | (–2.3) |
| | Phenol | Dibutylether | –24.0 | –5.74 | |
| **Oxygen-Nitrogen** | | | | | |
| O - H...N/–\ | Phenol | Triethylamine | –35.0 | –8.37 | –7.41 kcal/mol (–3.7) |
| O - H...N//\ | Phenol | Pyridine | –27.0 | –6.45 | –7.41 kcal/mol (–3.7) |
| N - H...O = C/\ | γ-Butyrolactam | γ-Butyrolactam | –14.5 | –3.47 | –3.94 kcal/mol |
| | α-Pyridone | α-Pyridone | –18.5 | –4.42 | (–2.0) |
| N - H...O/\ | Aniline | Tetrahydrofuran | –12.5* | –2.99 | |
| **Nitrogen-Nitrogen** | | | | | |
| N - H...N/–\ | Aniline | Aniline | –7.0* | –1.67 | |
| N - H...N//\ | Indole | Pyridine | –15.0 | –3.59 | –3.41 kcal/mol |
| | Pyrrole | Pyridine | –13.5 | –3.23 | (–1.7) |
| **Nitrogen-Sulfur** | | | | | |
| S – H...N//\ | Thiophenol | Pyridine | –10.0 | –2.39 | –3.08 kcal/mol (–1.5) |
| N – H...S = C//\ | γ-Thiobutyrolactam | γ-Thiobutyrolactam | –12.0 | –2.87 | –3.08 kcal/mol |
| | α-Thiopyridone | α-Thiopyridone | –14.5 | –3.47 | (–1.5) |
| N – H...S/\ | Thiocyanic acid | n-Butyl sulfide | –15.0 | –3.59 | –3.08 kcal/mol (–1.5) |
| **π-Oxygen, Nitrogen‡** | | | | | |
| O – H...π | Phenol | Hexamethyl benzene | –7.0 | –1.67 | –1.67 kcal/mol (–0.8) |
| N – H...π | Aniline | Benzene | –7.0* | –1.67 | –1.67 kcal/mol (–0.8) |

··· Denotes a hydrogen bond.

† Solvent system is tetrachloromethane in all cases apart from * which are for cyclohexane. These values are probably going to be significantly lower in a protein environment due to the presence of bound water molecules, which may bring values down by a factor of 2, which allows for the difference in dielectric constant.

‡ Calculations of hydrogen bond energies with aromatic rings have been reported by Levitt and Perutz[20].

*Notes:* The dielectric constant of $CCl_4$ is 2.238 at 293 K, whereas that of cyclohexane is 2.023, and both decrease with an increase in temperature such that they will be about 2.2 at 310 K. However, globular protein interiors are thought to possess a dielectric constant of ~4. The average hydrogen bond energy for interactions within proteins is therefore likely to be about –2 kcal/mol. For more precise estimations, however, one would need to distinguish between O...O, O...N, and N...N hydrogen bonds, as can be appreciated from the above values in parentheses which would range from –0.8 to –3.7 kcal/mol with that of amide hydrogen bonds representing the average value of –2 kcal/mol.
